# MFR-DTA: a multi-functional and robust model for predicting drug–target binding affinity and region

**DOI:** 10.1093/bioinformatics/btad056

**Published:** 2023-01-27

**Authors:** Yang Hua, Xiaoning Song, Zhenhua Feng, Xiaojun Wu

**Affiliations:** School of Artificial Intelligence and Computer Science, Jiangnan University, Wuxi 214122, China; School of Artificial Intelligence and Computer Science, Jiangnan University, Wuxi 214122, China; School of Computer Science and Electronic Engineering, University of Surrey, Guildford GU2 7XH, UK; School of Artificial Intelligence and Computer Science, Jiangnan University, Wuxi 214122, China

## Abstract

**Motivation:**

Recently, deep learning has become the mainstream methodology for drug–target binding affinity prediction. However, two deficiencies of the existing methods restrict their practical applications. On the one hand, most existing methods ignore the individual information of sequence elements, resulting in poor sequence feature representations. On the other hand, without prior biological knowledge, the prediction of drug–target binding regions based on attention weights of a deep neural network could be difficult to verify, which may bring adverse interference to biological researchers.

**Results:**

We propose a novel Multi-Functional and Robust Drug–Target binding Affinity prediction (MFR-DTA) method to address the above issues. Specifically, we design a new biological sequence feature extraction block, namely BioMLP, that assists the model in extracting individual features of sequence elements. Then, we propose a new Elem-feature fusion block to refine the extracted features. After that, we construct a Mix-Decoder block that extracts drug–target interaction information and predicts their binding regions simultaneously. Last, we evaluate MFR-DTA on two benchmarks consistently with the existing methods and propose a new dataset, sc-PDB, to better measure the accuracy of binding region prediction. We also visualize some samples to demonstrate the locations of their binding sites and the predicted multi-scale interaction regions. The proposed method achieves excellent performance on these datasets, demonstrating its merits and superiority over the state-of-the-art methods.

**Availability and implementation:**

https://github.com/JU-HuaY/MFR.

## 1 Introduction

Drug–target interaction (DTI) prediction is crucial to drug discovery, and computer-assisted DTI has become the most popular and efficient approach for the task (Hua *et al.*, 2022; Tian *et al.*, 2016). The existing mainstream DTI methods (Cheng *et al.*, 2012; Jacob and Vert, 2008; Wang and Zeng, 2013) are all machine-learning based. These methods mainly include three steps: drug and protein feature extraction, interaction information refinement and classification (Chen *et al.*, 2018; Tian *et al.*, 2016; Yamanishi *et al.*, 2010). In general, DTI prediction is formulated as a binary classification task. However, the use of a binary label (0 or 1) is challenging to reflect the interaction intensity quantitatively. To bridge this gap, Tang *et al.* (2014) firstly considered DTI prediction as a regression task and proposed to use the Regularized Least-Squares method with Kronecker kernels as a solver. Then, He *et al.* (2017) used gradient booster (Singh and Gupta, 2014; Svetnik *et al.*, 2005) to improve the performance of the learning-based methods and proposed the concept of predicting Drug–Target binding Affinities (DTAs). Binding affinity is closely related to dissociation constant ($K_{d}$), inhibition constant ($K_{i}$), or the half-maximal inhibitory concentration ($IC_{50}$) (He *et al.*, 2017), and the low values of these indexes ($K_{d}$, $K_{i}$ and $IC_{50}$) usually indicate high affinity (Cer *et al.*, 2009). Therefore, most researchers use the negative logarithm ($pK_{d}$ and $pK_{i}$) of these indicators to describe binding affinity.

With the success of deep learning, a variety of deep networks have been studied for DTI and DTA prediction. Öztürk *et al.* (2018) first proposed the DeepDTA model using biological sequence features extracted by a Convolution Neural Network (CNN) to predict DTA and demonstrated promising results. Further, Lee *et al.* (2019) proposed to use molecular fingerprints as drug features and achieved better prediction accuracy than the use of sequence features in DeepDTA. Following this discovery, they replaced CNN with the Multi-Layer Perceptron (MLP) to extract drug fingerprints and developed an optimized DeepConvDTI model for DTI prediction. Inspired by DeepDTA, Abbasi *et al.* (2020) proposed DeepCDA and applied the Long Short-Term Memory (LSTM) (Hochreiter and Schmidhuber, 1997) layer after each CNN layer to improve the model capability in capturing the information among biological sequences. More importantly, DeepCDA further interpreted the model by predicting the interaction binding regions (BRs) through the weight matrix in the attention mechanism. With the wide use of graph networks, Tsubaki *et al.* (2019) demonstrated that the use of molecular structure graphs in representing molecular characteristics could further improve the performance of a DTI prediction model in terms of accuracy. On this basis, Chen *et al.* (2020) proposed TransformerCPI based on the widely used Transformer framework (Vaswani *et al.*, 2017). Although this method further broadened the vision of deep learning in drug–protein interaction prediction, the extensive memory consumption of the self-attention mechanism limits its practical applications. To mitigate this issue, CPInformer (Hua *et al.*, 2022) used a more efficient model, namely Informer (Zhou *et al.*, 2021), to replace Transformer for drug–protein interaction prediction. Besides, CPInformer demonstrates that fingerprints (Lee *et al.*, 2019) can assist graph features (Chen *et al.*, 2020) to alleviate the challenge of distinguishing similar drug structures.

**Fig. 1.**
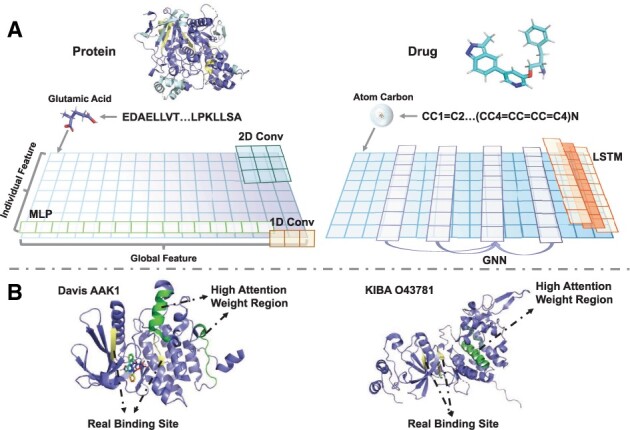
The sub-figure (**A**) demonstrates the receptive fields of the existing molecular feature extraction methods, and sub-figure (**B**) presents the predicted attention weights and real binding sites of two examples: protein ‘AAK1’ from the Davis dataset and protein ‘O43781’ from the KIBA dataset

Although most existing DTI and DTA prediction methods have achieved promising results, they are not without problems. On the one hand, the mainstream biological sequence feature extraction methods, including CNN (Öztürk *et al.*, 2018, 2019; Zhao *et al.*, 2019), MLP (Lee *et al.*, 2019; Shin *et al.*, 2019), LSTM (Abbasi *et al.*, 2020; Mukherjee *et al.*, 2022), GNN (Lin *et al.*, 2020; Nguyen *et al.*, 2021; Tsubaki *et al.*, 2019; Zheng *et al.*, 2020) and Transformer-based backbone (Chen *et al.*, 2020; Hua *et al.*, 2022), have inadequacies in extracting rich protein and drug features. The elements of a protein or a drug, such as amino acids and atoms, influence the prediction task significantly. As shown in Figure 1A, both 1D convolution and MLP completely ignore the individual features of each element. In contrast, LSTM and GNN extract individual features directly, but they are insufficient in obtaining global features. While 2D convolution extracts both individual and global features by increasing convolution kernels or stacking more convolution layers, its computational consumption grows rapidly. Besides, the Transformer-based backbone is overly redundant in parameters, making it less friendly for efficient protein and drug feature extraction. On the other hand, many existing approaches (Abbasi *et al.*, 2020; Chen *et al.*, 2020; Hua *et al.*, 2022; Tsubaki *et al.*, 2019) try to improve their performance via attention mechanisms. However, these methods attempt to identify the BRs via high-attention responses, which is difficult to verify and lacks a theoretical basis. This is because the highlighted region is not linked with the biological characteristics of the protein. As shown in Figure 1B, the predicted BRs by these methods are often contrary to the real ones. This may mislead biomedical researchers in locating the binding sites.

To mitigate the above issues, we propose a novel Multi-Functional and Robust Drug–Target binding Affinity prediction (MFR-DTA) model, which has three main innovations. First, we develop a new biological sequence feature extraction block, namely BioMLP/CNN, that contains a global feature extractor and an individual feature extractor. To effectively extract global (reflecting elements arrangement information) and individual (expressing sequence composition information) features, BioMLP/CNN processes biological sequence features from two dimensions of the input matrix. Moreover, we use a spatial attention block to capture the local relationship among the adjacent elements, further enriching the extracted individual features. By design, the proposed block provides effective and informative biological sequence feature extraction with fewer model parameters by integrating global and individual information. Second, we propose a new Elem-feature fusion block to further refine the protein and drug features. We represent the biological sequence features of proteins and drugs from two aspects. Specifically, for protein feature representation, we use amino acid embedding (AAE) and word embedding (WE), and for drugs, we use FCFPs and GNN features. The fusion block generates attention matrices by combining the two types of features to ensure that the complementary elements of each type of features are highlighted. Therefore, the proposed Elem-feature fusion block obtains comprehensive features from different representations of a drug or a protein. Third, we construct a Mix-Decoder block, which can predict drug–target BRs and extract interaction feature vectors simultaneously. On the one hand, unlike the previous methods (Abbasi *et al.*, 2020; Chen *et al.*, 2020; Hua *et al.*, 2022; Tsubaki *et al.*, 2019) that use high-attention responses as predicted regions, we introduce the protein binding site information for supervised learning. We first sample the drug feature matrix as a convolution kernel and multiply the protein features with the kernel via the convolution operation. Then, we record the convolution results as the drug–target response vector and set the region with high values in the response vector as the BR. On the other hand, we expand the response vector into the BR information matrix to weigh the protein features and fuse the drug Adj information to enrich drug features. Then, we extract interaction features between a protein and a drug via two self-enhancement (S-E) blocks and a cross-attention (C-A) block, which stabilizes the convergence and improves the performance of a trained model. Last, the model predicts binding affinity by applying fully connected layers to the drug–protein interaction features.

We evaluate the proposed MFR-DTA method on two benchmarking datasets. The experimental results demonstrate that MFR-DTA achieves superior performance over the state-of-the-art methods. However, the existing benchmarks do not have a specified evaluation metric for the performance of a BR predictor on the sequence format data, such as protein sequence and Simplified Molecular-Input Line-Entry System of drugs. Hence, we take the probability of protein binding sites falling in the prediction region as the measurement to evaluate the prediction accuracy. Moreover, we use a new dataset, sc-PDB (Gaber *et al.*, 2019), to evaluate the performance of the proposed method in BR prediction of unseen samples. As compared with the existing methods (Abbasi *et al.*, 2020; Chen *et al.*, 2020; Hua *et al.*, 2022; Tsubaki *et al.*, 2019), the proposed method performs better in accuracy. Besides, we visualize some samples to intuitively reflect the relationships between the actual binding sites and the predicted ones.

In summary, the main contributions of MFR-DTA include:

- A BioMLP/CNN block for rich protein and drug feature extraction. To extract individual features and associated features of an element in a sequence simultaneously, BioMLP/CNN is the first to extract individual features of biological sequence elements.
- An Elem-feature fusion block for effective feature mining. The aim is to refine comprehensive drug and protein features. It can effectively maintain the core information of the two aspects.
- A Mix-Decoder block for drug–target BR estimation, which extracts DTI features effectively and predicts their BRs simultaneously.
- A new dataset, sc-PDB, for further evaluation of the proposed method in predicting drug–target BRs.

The rest of this article is organized as follows. We first introduce the proposed MFR-DTA method in Section 2. Then, we report the experimental results in Section 3 and draw the conclusion in Section 4.

## 2 The proposed MFR-DTA method

**Fig. 2.**
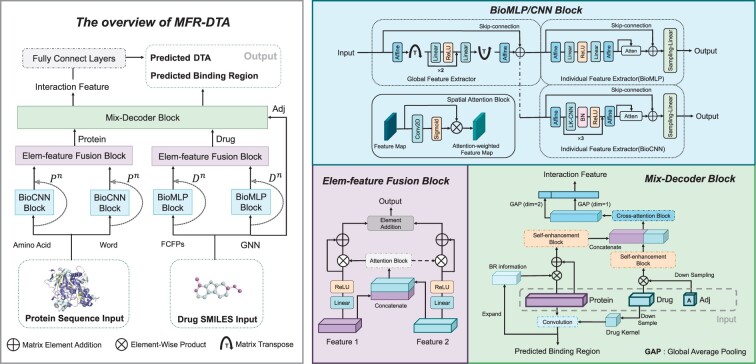
The proposed MFR-DTA method for joint DTA and site prediction. The overall pipeline is shown on the left-hand side. The proposed method has three innovations: the BioMLP/CNN blocks, the Elem-feature fusion block and the Mix-Decoder block, which are demonstrated on the right-hand side

The overall pipeline of the proposed MFR-DTA method is shown in Figure 2. Our method has three main innovative blocks: BioMLP/CNN, Elem-feature fusion and Mix-Decoder. Besides, MFR-DTA uses fully connected layers to predict DTAs using the interaction features extracted by the Mix-Decoder block. In the rest of this section, we will introduce these three components in more detail.

### 2.1 The BioMLP/CNN block

We design BioMLP and BioCNN by inheriting the ResMLP block (Touvron *et al.*, 2022) to extract drug and protein features, respectively. As shown in Figure 2, the input of a BioMLP/CNN block is either drug or protein feature representations. To be specific, the drug feature representations consist of FCFPs and GNN features (Hua *et al.*, 2022), and the protein feature representations contain AAE and WE (Hua *et al.*, 2022; Öztürk *et al.*, 2018). These representations include both global and individual features (such as amino acid features in proteins and atom features in drug structures). To extract the above two features, the BioMLP/CNN blocks adopt two sub-modules, i.e. the global and individual feature extractors, according to the following strategy.

First, we use the global feature extractor to extract the correlation of different biological sequences. Note that, we use the same architecture for the global feature extractors of BioCNN and BioMLP. The global feature extractor consists of affine blocks (Touvron *et al.*, 2022), fully connected layers and ReLU activation layers. The architecture can be formulated as:

(1) $$\begin{array}{c} X_{\mathrm{out}}=X_{in}+AF(FC_{\left(3\right)}{\left(AF{\left(X_{in}\right)}^{T}\right)}^{T}) \end{array},$$

where $X_{in}\in R^{L\times C}$ and $X_{\mathrm{out}}\in R^{L\times C}$ are the input and the output of the global feature extractor, *L* is the length of drug or protein features, *C* is the embedding channel size. The affine block is defined as AF(X)=Diag(α)X+β, where Diag() creates a diagonal matrix, α and β are trainable weighting vectors. $FC_{\left(3\right)}$ consists of three fully connected layers and two ReLU layers. Besides, we apply skip connections to BioMLP/CNN as shown in Figure 2.

Second, we use two individual feature extractors to further mine the biological sequence composition information, as constituent element individual features, according to the output global features. The two extractors in the BioMLP/CNN blocks are different:

(2) $$\begin{array}{c} X_{\mathrm{MLP}}=FC(X_{\mathrm{out}}+\mathrm{Att}(AF(FC_{\left(2\right)}(AF(X_{\mathrm{out}}))))) \end{array},$$

(3) $$\begin{array}{c} X_{\mathrm{CNN}}=FC(X_{\mathrm{out}}+\mathrm{Att}(AF(CNN_{LK}(AF(X_{\mathrm{out}}))))) \end{array},$$

(4) $$\begin{array}{c} \mathrm{Att}(X)=X*\sigma (CNN_{2D}(X)) \end{array},$$

where $X_{\mathrm{MLP}}\in R^{L_{D}\times C_{D}}$ and $X_{\mathrm{CNN}}\in R^{L_{P}\times C_{P}}$ are the output features of BioMLP and BioCNN, $L_{D/P}$ and $C_{D/P}$ are the length and channel size of drug/protein features. FC() is the fully connected layer. In BioMLP, $FC_{\left(2\right)}$ contains two fully connected layers and one ReLU function layer. In BioCNN, $CNN_{LK}$ contains three convolution blocks, each consisting of a large-kernel group 1D convolution layer, a batch normalization layer and a ReLU layer. Additionally, the spatial attention module, Att(), captures the local relationship among adjacent elements through 2D convolution $CNN_{2D}$ and normalizes the captured information by the Sigmoid function, σ(), to enrich the individual features.

Last, the BioMLP/CNN block combines global and individual features via the addition operation and uses a fully connected layer to obtain comprehensive and representative features. Specifically, BioMLP uses fewer linear layers to extract the individual features, improving its efficiency and being suitable for shorter biological sequences, such as drug sequences. In contrast, BioCNN uses more large-kernel convolution layers, effectively extracting the features of complex sequences, such as protein sequences. Besides, the number of cascading feature extraction modules also influences the model prediction accuracy. Hence, we determine the optimal number of BioMLP and BioCNN blocks ($D^{n}$ & $P^{n}$) based on the experimental results and present them in Table 1.

**Table 1.** Hyper-parameter settings of MFR-DTA

Hyper-parameter	Davis	KIBA
Kernel size of $CNN_{LK}$	(7, 15, 31)	(7, 23, 35)
Kernel size of $CNN_{2D}$	7	3
$P^{n}$	3	2
$D^{n}$	2	3
$L_{D}$, $L_{P}$	51, 1200	101, 1200
$C_{P}$, $C_{D}$, $C_{S}$	100, 75, 75	–

### 2.2 The Elem-feature fusion block

We represent the biological sequence features of proteins and drugs from different perspectives. To be specific, we adopt AAE and WE for protein feature representations, and FCFPs and GNN for drug feature representations. However, it is vital to fuse and refine them to represent the biological sequence features so that to further improve the accuracy of the proposed model. To enrich the semantic information of the refined features and balance the contribution of different feature types, we propose a fusion block as shown in Figure 2. The block can transform the feature matrices, $X_{1}\in R^{L\times C_{S}}$ and $X_{2}\in R^{L\times C_{S}}$, into the fused feature matrix, $X_{f}\in R^{L\times C_{S}}$, in which $C_{S}$ is the channel of drug and protein features extracted by the BioMLP/CNN block:

(5) $$\begin{array}{c} X_{f}=FC_{\left(1\right)}(X_{1})*W_{\mathrm{att}}+X_{1}+FC_{\left(1\right)}(X_{2})*(1-W_{\mathrm{att}})+X_{2} \end{array},$$

(6) $$\begin{array}{c} W_{\mathrm{att}}=\sigma (CNN_{2D}(\mathrm{Concat}(X_{1},X_{2}))) \end{array},$$

where $FC_{\left(1\right)}$ consists of a fully connected layer and a ReLU function layer, * is the element-wise product. Concat is the concatenation operation, and the 2D convolution operation ($CNN_{2D}$) extracts the local features from the concatenated feature map. Besides, we use the Sigmoid function, σ(), to normalize the local features to obtain the attention weight matrix $W_{\mathrm{att}}$ of the feature $X_{1}$ and set $\left(1-W_{\mathrm{att}}\right)$ as the attention weight matrix of the feature $X_{2}$ to promote both feature types that could complement each other. The residual connection is also used in the proposed feature fusion block. Last, we combine the two features using the addition operator.

### 2.3 The Mix-Decoder block

The Mix-Decoder block plays a crucial role in our method, predicting BRs and extracting interaction features simultaneously. As shown in Figure 2, the input of this block contains three components: refined drug features, refined protein features and drug Adj matrices. To predict drug–target BRs, we first obtain the drug kernel $K_{d}\in R^{C_{S}\times C_{S}}$ by sampling the drug features $F_{d}\in R^{L_{D}\times C_{S}}$ with a linear layer. Then, we get the drug–target response vector $s\in R^{L_{P}\times 1}$ by filtering the protein features $F_{p}\in R^{L_{P}\times C_{S}}$ with the drug kernel $K_{d}$:

(7) $$\begin{array}{c} s_{i}=\sum_{m=0}^{c}\sum_{n=0}^{c}K_{d}(m,n)*F_{p}(i-m,j-n) \end{array}$$

where $s_{i}$ is the *i*th element of the response vector, * stands for element-wise product. In the response vector, the elements with the highest values are recognized as the drug–target BRs.

To extract DTI features, we first extend the drug–target response vector, **s**, to the BR information matrix with the size of $L_{P}\times C$ via repetitive padding. Then, we apply element-wise multiplication to the protein features with the BR information to encourage our model to focus on the BR. Furthermore, we down-sample the Adj matrix into an atom connectivity vector via global average pooling and expand the connectivity vector into the Adj information matrix, $M_{c}\in R^{L_{D}\times C_{S}}$, via repetitive padding. Hence, the refined drug feature, $X_{d}\in R^{L_{D}\times C_{S}}$, can be highlighted on the feature aggregation region by element-wisely multiplying the Adj information matrix, $M_{c}$. After that, the S-E block is proposed to enhance the drug and protein features:

(8) $$\begin{array}{c} X_{\mathrm{out}}=X_{in}W_{1}*f_{a}(X_{in}) \end{array},$$

(9) $$\begin{array}{c} f_{a}(X)=\mathrm{Softmax}(\frac{XW_{m}}{\sqrt{C_{S}}}) \end{array},$$

where $X_{in}\in R^{L\times C_{S}}$ and $X_{\mathrm{out}}\in R^{L\times C_{S}}$ are the input and output of the S-E block, *L* is the length of the drug or protein features and $C_{S}$ is the channel size of the drug and protein features refined by the Elem-feature fusion block. $W_{1}$ and $W_{m}\in R^{C_{S}\times C_{S}}$ are parameter matrices. The linear normalized function, $f_{a}()$, enhances the regions that have a significant influence on the model performance in the feature matrix X.

We also design a C-A block to extract the drug–protein interaction features:

(10) $$\begin{array}{c} Y_{\mathrm{out}}=X_{m}+{\left(X_{m}W_{m1}\right)}^{T}W_{m2}*f_{a}(X_{m}) \end{array},$$

(11) $$\begin{array}{c} X_{m}=\mathrm{Concat}(X_{p},X_{d}) \end{array},$$

where $Y_{\mathrm{out}}\in R^{(L_{P}+L_{D})\times C_{S}}$ is the output of the C-A block. $W_{m1}\in R^{C_{S}\times C_{S}}$ and $W_{m2}\in R^{\left(L_{P}+L_{D})\times (L_{P}+L_{D}\right)}$ are parameter matrices. $X_{p}\in R^{L_{P}\times C_{S}}$ and $X_{d}\in R^{L_{D}\times C_{S}}$ are the feature matrices of a protein and a drug. Concat is the concatenation operation. Both the S-E and C-A blocks can improve the performance of our model effectively, and we will discuss their impacts in Section 3.2.3. To improve the representation capability of the output features, we sample $Y_{\mathrm{out}}$ from two dimensions with global average-pooling and concatenate them as the interaction features, $V_{\mathrm{out}}\in R^{\left(L_{P}+L_{D}+C_{S}\right)}$.

### 2.4 Loss functions and hyper-parameter settings

For affinity prediction, we follow DeepDTA (Öztürk *et al.*, 2018) and use the mean square error (MSE) loss, $\mathrm{MSE}=\frac{1}{n}\sum_{i=1}^{n}{\left(P_{i}-Y_{i}\right)}^{2}$, where $P_{i}\in R^{B\times 1}$ and $Y_{i}\in R^{B\times 1}$ are the predicted affinity value and the affinity label of the *i*th sample, *B* is the batch size.

We collect the binding site information of all the proteins in the Uniprot (UniProt Consortium, 2019) dataset and embed them as label vectors with the size of $L_{P}\times 1$. In label embedding, we code an element in non-BRs as 0 and an element in BRs as the affinity value to match the DTI strength. Unfortunately, the BR is a tiny part of the label vector, and its position is diverse. The sparsity of the label vector leads to excessive outliers, but the MSE loss is sensitive to outliers. To alleviate this issue, we adopt the Rectified Wing (RWing) loss function (Feng *et al.*, 2020) to train the model for BR prediction. RWing loss inherits the ability of Wing loss (Feng *et al.*, 2018), showing excellent robustness to various ranges of loss calculations. Additionally, RWing loss omits tiny errors by rectifying the loss function around zero to reduce the impact of manual annotation noise on the training of a network. The loss function is defined as:

(12) $$\begin{array}{c} \mathrm{RWing}(x)=\{\begin{array}{cc} 0 & \;\text{if}\;|x|<r\\ w\;\ln(1+(|x|-r)/\epsilon ) & \;\text{if}\;r\leq |x|<w\\ |x|-C & \;\text{otherwise}\; \end{array} \end{array},$$

where the non-negative parameter *r* sets the range of rectified region to (−r,r) for very small values. For small-medium range values with the absolute value in [r,w), RWing uses a modified logarithm function, where ϵ limits the curvature of the non-linear region and C=w−w ln(1+(|x|−r)/ϵ) is a constant that smoothly links the linear and non-linear parts. The MFR-DTA model is trained in a multi-task manner by jointly using the MSE and RWing loss functions as the final loss.

The proposed method is implemented in Python 3.8 with PyTorch 1.8.0. The experiments are carried out on a machine with Ubuntu 20.04, Intel Core i7-11700K CPU and one NVIDIA GeForce RTX 3090 card. We use the AdamW optimizer (Loshchilov and Hutter, 2018) for network training. In the training process, we set the learning rate as $5\times 10^{-4}$, the weight decay as $1\times 10^{-3}$, the batch size, *B*, as 16 and the dropout ratio as 0.1. We set *w* as 1, *r* as 3 and ϵ as 0.15 in the RWing loss function. More hyper-parameter settings are listed in Table 1.

## 3 Experimental results

In this section, we first introduce the benchmarking datasets used for the evaluation, as well as the evaluation metrics. Then, the effectiveness of each proposed innovative component is analysed in the ablation study. After that, we compare the proposed method with the state-of-the-art approaches on all the benchmarks. Last, we further visualize the capability of the proposed method in predicting drug-targeted BRs.

### 3.1 Evaluation datasets and metrics

**Fig. 3.**
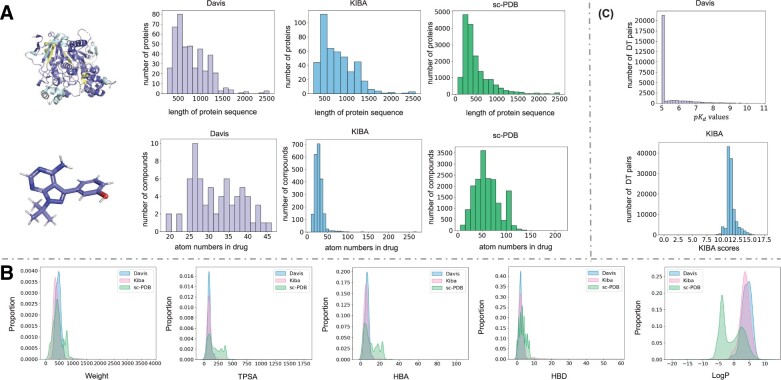
The sub-figure (**A**) presents the protein length and atom numbers of the drugs in Davis, KIBA and sc-PDB. The sub-figure (**B**) presents the distribution of the molecular properties, including weights, TPSA, the number of HBD, the number of HBA and the oil–water partition coefficient: LogP, of the datasets. The sub-figure (**C**) presents the binding affinity values in the Davis and KIBA datasets

To be consistent with DeepDTA (Öztürk *et al.*, 2018), we evaluate our model on two benchmarks, Davis (Davis *et al.*, 2011) and KIBA (Tang *et al.*, 2014). Additionally, we convert the data of a new 3D dataset, sc-PDB (Gaber *et al.*, 2019), into the sequence format to evaluate the performance of the proposed method for BR prediction. The statistics for the three datasets are shown in Table 2. And we present the chemical information of the datasets in Figure 3. The protein sequence lengths of the three datasets are mainly <1500, with a relatively positive distribution, and most drugs are small molecules consisting of <100 atoms. However, the number of drugs that have 50+ atoms in the sc-PDB dataset is more than those of the other two datasets. Also, the properties of drugs in Davis and KIBA are similar, including weights, topological polar surface areas (TPSA), the number of hydrogen-bonded donors (HBD), the number of hydrogen-bonded acceptors (HBA) and the oil–water partition coefficient: LogP, and these properties determine whether and how a drug interacts with a protein. But their properties are also different from sc-PDB, so we can evaluate our model using the sc-PDB to predict the drug–target BR of unseen samples. Besides, Figure 3C depicts the label distributions of the two datasets. It is clear that both pose challenges for robust model training. The label distribution of Davis is unbalanced, and the label values aggregate at around 5. Although the distribution of KIBA is more balanced, the label values are also concentrated in the middle part. Both limit the generalization capability of a trained model on the datasets. Following the existing literature (He *et al.*, 2017; Öztürk *et al.*, 2018), we transform the dissociation constant value $K_{d}$ in Davis to the $pK_{d}$ value as the binding affinity value: $pK_{d}=-\text{log}\;10\left(\frac{K_{d}}{1e9}\right)$.

**Table 2.** Statistics of the benchmarking datasets

	Davis	KIBA	sc-PDB
No. proteins	442	229	4782
No. drugs	68	2111	6326
No. total samples	30 056	118 254	16 034
No. active samples	2457	22 729	–
No. inactive samples	27 599	95 525	–
No. train samples	25 046	98 545	–
No. test samples	5010	19 709	–

Following DeepDTA (Öztürk *et al.*, 2018), the training samples are further divided into training and validation sets via the 5-fold cross-validation. We use the hyper-parameters introduced in Section 2.4 for network training. Then, the test set is used to evaluate the five trained models and the average results are reported.

Besides, we evaluate the proposed model by using the concordance index (CI) (Gönen and Heller, 2005), MSE, $r_{m}^{2}$ index and area under the precision–recall curve (AUPR) metrics. To be specific, CI is calculated as $CI=\frac{1}{Z}\sum_{\delta_{i}>\delta_{j}}h\left(b_{i}-b_{j}\right)$, where $\delta_{i}$ and $\delta_{j}$ are the label values of the affinity of two samples. $\delta_{i}$ is larger than $\delta_{j}$. $b_{i}$ is the prediction value of the *i*th sample, $b_{j}$ is the prediction value of the *j*th sample, *Z* is the normalized constant and h(x) is the step function (Pahikkala *et al.*, 2015):

(13) $$\begin{array}{c} h(x)=\{\begin{array}{cc} 1, & \;\text{if}\;x>0\\ 0.5, & \;\text{if}\;x=0\\ 0, & \;\text{if}\;x<0 \end{array} \end{array}.$$

Further, we use the $r_{m}^{2}$ metric proposed by DeepDTA (Öztürk *et al.*, 2018) to measure the external prediction performance of a method, where $r_{m}^{2}=r^{2}*(1-\sqrt{r^{2}-r_{0}^{2}})$, $r^{2}$ and $r_{0}^{2}$ are the squared correlation coefficient values between the measured and predicted values with and without intercept, respectively. The higher the $r_{m}^{2}$ value of the model on the test set, the better its performance. Additionally, we use AUPR to evaluate the performance of a model trained on unbalanced data. To measure AUPR reasonably, the quantitative datasets are converted into binary datasets by selecting binding affinity thresholds. For the Davis dataset, we set the $pK_{d}$ value to seven as the threshold ($pK_{d}$>7 binds) by following Öztürk *et al.* (2018). For the KIBA dataset, we use the suggested threshold value of 12.1 (He *et al.*, 2017; Öztürk *et al.*, 2018; Tang *et al.*, 2014).

### 3.2 Ablation study

For the ablation study, we first analyse the effectiveness of each innovative element in MFR-DTA. Then, we investigate different protein feature selection methods. Last, we compare the proposed Mix-Decoder block with other interaction feature extraction methods.

#### 3.2.1 Analysis of different innovative elements

In this part, we use CPInformer (Hua *et al.*, 2022) as our baseline method. CPInformer represents the protein primary structure sequence by WE. It also uses a molecular feature fusion module to fuse and refine the FCFPs and GCN features of drugs. Then, CPInformer uses the Informer model (Zhou *et al.*, 2021) for DTI feature extraction and predicts DTI via fully connected layers. In this article, we improve the baseline method by adding our BioMLP/CNN, Elem-feature fusion and Mix-Decoder blocks. We report the results of the baseline model and the new models with different configurations in Table 3.

**Table 3.** The ablation study results obtained on the KIBA and Davis datasets, bold: best results

Dataset	Model	BioMLP/CNN	Elem-feature fusion	Mix-Decoder	CI↑	MSE↓	$r_{m}^{2}$ ↑	AUPR↑
Davis	Baseline	–	–	–	0.874 ± 0.002	0.277 ± 0.003	0.618 ± 0.004	0.663 ± 0.003
	Model-1	√	–	–	0.887 ± 0.004	0.254 ± 0.004	0.648 ± 0.003	0.695 ± 0.002
	Model-2	–	√	–	0.879 ± 0.003	0.269 ± 0.005	0.625 ± 0.004	0.652 ± 0.005
	Model-3	√	√	–	0.894 ± 0.001	0.239 ± 0.003	0.653 ± 0.002	0.719 ± 0.005
	Model-4	–	–	√	0.881 ± 0.004	0.246 ± 0.003	0.649 ± 0.005	0.703 ± 0.003
	MFR-DTA	√	√	√	**0.905 ± 0.001**	**0.221 ± 0.001**	**0.705 ± 0.003**	**0.751 ± 0.003**
KIBA	Baseline	–	–	–	0.867 ± 0.003	0.183 ± 0.002	0.677 ± 0.002	0.773 ± 0.004
	Model-1	√	–	–	0.877 ± 0.002	0.162 ± 0.003	0.679 ± 0.004	0.787 ± 0.002
	Model-2	–	√	–	0.869 ± 0.004	0.172 ± 0.004	0.683 ± 0.005	0.801 ± 0.003
	Model-3	√	√	–	0.886 ± 0.006	0.159 ± 0.005	0.703 ± 0.002	0.812 ± 0.002
	Model-4	–	–	√	0.881 ± 0.003	0.167 ± 0.004	0.709 ± 0.003	0.792 ± 0.005
	MFR-DTA	√	√	√	**0.898 ± 0.002**	**0.136 ± 0.001**	**0.789 ± 0.002**	**0.827 ± 0.003**

The table demonstrates that our BioMLP/CNN module (Model-1) improves the performance of the baseline method because the proposed module extracts more comprehensive features (including the individual and global features) that are essential for accurate and reliable DTA prediction. According to the results obtained by Model-2, the proposed Elem-feature Fusion block can better fuse the drug and protein features as compared with the fusion method in the baseline approach. By combining the above two innovative elements, we can see that the performance of Model-3 is further improved. To be specific, Model-3 achieves 0.894 and 0.886 in CI on the Davis and KIBA datasets, much better than the baseline method. This experiment also verifies the superiority of the proposed feature extraction and fusion blocks.

To validate the effectiveness of the Mix-Decoder block, we replace the original ProbSparse self-attention module in the baseline method with the Mix-Decoder block, denoted as Model-4. The Mix-Decoder block introduces prior knowledge of protein BRs into the model so it improves the performance of the baseline method. The performance of Model-4 has been improved by 3.1% and 1.6% on Davis and KIBA, respectively, in terms of MSE. The results initially demonstrate the efficiency of our Mix-Decoder block, and we will further discuss the main contribution of this block in Section 3.2.3. Last, we combine the three innovative elements to construct our final MFR-DTA method. As compared with the baseline method as well as the other configurations, the combination of all the proposed innovative components achieves the best results on both datasets.

#### 3.2.2 Analysis of protein feature extraction and fusion approaches

CPInformer (Hua *et al.*, 2022) has demonstrated that the fusion of graph and fingerprint features outperforms the single use of them, so we do not demonstrate this again here. To further clarify our design, we analyse the effectiveness of different protein feature representation and fusion approaches. We use two protein feature representations, including AAE and WE. The results are reported in Table 4. It is clear that both feature representation methods achieve good performance on the two datasets. The biological properties of AAE assist the proposed model in predicting the affinity trend so it performs better in terms of the CI metric. In contrast, the WE feature extraction method provides more semantic information, encouraging the model to converge and perform better in MSE.

**Table 4.** A comparison of different protein feature extraction and fusion approaches, evaluated in terms of CI and MSE, bold: best results

Dataset	Representation	Approaches	CI↑	MSE↓
Davis	AAE	–	0.891 ± 0.003	0.252 ± 0.005
	WE	–	0.889 ± 0.004	0.246 ± 0.003
	AAE&WE	Concat	0.894 ± 0.003	0.253 ± 0.003
	AAE&WE	Concat&CNN	0.896 ± 0.005	0.241 ± 0.004
	AAE&WE	CPInformer	0.896 ± 0.002	0.239 ± 0.003
	AAE&WE	ours	**0.905 ± 0.001**	**0.221 ± 0.001**
KIBA	AAE	–	0.883 ± 0.005	0.164 ± 0.004
	WE	–	0.881 ± 0.003	0.153 ± 0.004
	AAE&WE	Concat	0.889 ± 0.003	0.149 ± 0.005
	AAE&WE	Concat&CNN	0.888 ± 0.004	0.151 ± 0.003
	AAE&WE	CPInformer	0.891 ± 0.003	0.147 ± 0.004
	AAE&WE	ours	**0.898 ± 0.002**	**0.136 ± 0.001**

To verify whether the model performs better when introducing both representations, we adopt four fusion approaches to combine the above two features and refine the protein and drug features. These approaches include concatenation, convolution after concatenation, the fusion approach of CPInformer and the proposed Elem-feature fusion block. We report the results in Table 4, and the performance of the four fusion features outperforms that of a single one, further demonstrating that the two protein feature representations can complement each other. Further, the proposed feature fusion block demonstrates its superiority over the other three approaches on the two metrics in both benchmarks, proving its good feature fusion capability.

#### 3.2.3 Analysis of different interaction feature extraction methods

**Fig. 4.**
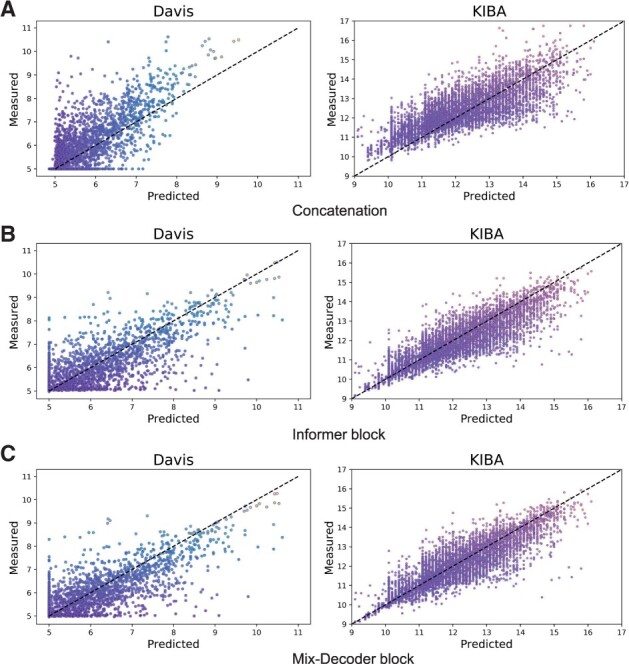
The performance of three interaction methods. The *X*- and *Y*-axis indicate the predicted and measured DTAs of a sample

In this part, we compare the concatenation (Lee *et al.*, 2019; Öztürk *et al.*, 2018; Tsubaki *et al.*, 2019) and the attention-based Informer block (Hua *et al.*, 2022) with our approach to further verify our Mix-Decoder block. We first replace the Mix-Decoder block with concatenation in MFR-DTA, resulting in 0.871 ± 0.004 (CI) and 0.272 (MSE) on Davis and 0.862 ± 0.007 (CI) and 0.197 (MSE) on KIBA. Then, we evaluate the performance of the Informer and Mix-Decoder blocks, as Model-3 and Model-5 in Table 3, respectively. To be more clear, we draw the scatter plot of the samples predicted by the above three models. As shown in Figure 4, the *X*- and *Y*-axis indicate the coordinates of predicted and measured affinities of a sample. On the Davis dataset, the number of samples with small label values is much more than those with larger values, so the affinities predicted by the model with concatenation are generally smaller than the measured ones. Without a well-designed mechanism, the model hastily falls into the trap of unbalanced convergence. In contrast, both the Informer and Mix-Decoder blocks can effectively alleviate this issue, while the performance of the Mix-Decoder block is more prominent. Besides, the label distribution of KIBA is relatively normal. The scatters of the three approaches concentrate on the diagonal moderately, while the scatters of the Mix-Decoder block are more concentrated.

**Fig. 5.**
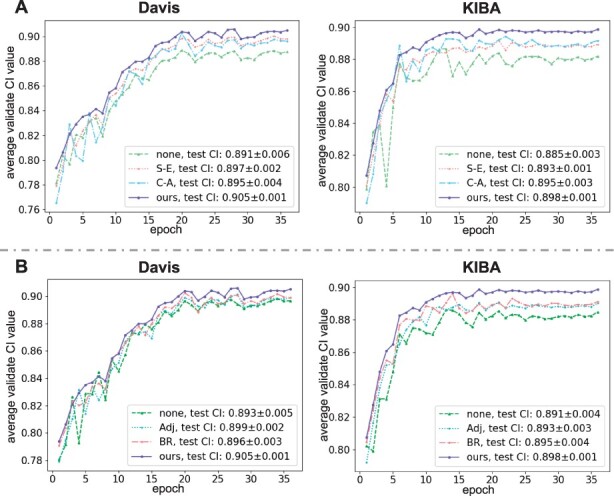
The sub-figure (**A**) presents the model performance on the validation set as the epoch increases with the addition of S-E and C-A blocks. The sub-figure (**B**) presents the model performance on the validation set as the epoch increases, fusing the Adj and the BR information

Furthermore, we analyse the main components involved in Mix-Decoder and display the corresponding experimental results in Figure 5. Note that, the curve represented by ‘none’ means that the model does not add the S-E and C-A blocks (in Fig. 5A) or does not fuse the Adj and BR information (in Fig. 5B). Figure 5A reveals that the S-E and C-A blocks improve the prediction accuracy. In detail, the fluctuation range of the curves which contain the S-E block reduces significantly, demonstrating that the S-E block can help the model converge more stably. Moreover, the model can combine both advantages by adopting the two blocks simultaneously. Figure 5B also demonstrates that the fusion of both information facilitates model training, especially when both are fused concurrently, and the model with fused features achieves the best result.

### 3.3 Comparison with the state-of-the-art methods

We compare the proposed MFR-DTA method with the existing mainstream DTA prediction models on the two benchmarks in Table 5. To be consistent with Section 3.2.1, we use the CI, MSE, $r_{m}^{2}$ index and AUPR as the evaluation metrics. We first compare our MFR-DTA model with the existing mainstream DTA prediction methods on the Davis dataset in Table 5. In this dataset, the unbalanced label distribution causes the predicted affinities of most models to be biased towards a smaller value and creates an obstacle for these models to perform well in terms of MSE. However, the proposed MFR-DTA method can achieve 1.2%, 0.8% and 2.3% performance gains in terms of CI, MSE and $r_{m}^{2}$ index, as compared with the second-best methods, i.e. DeepGLSTM and MATT-DTI. Besides, compared with DeepCDA, our method still achieves 1.5% performance improvement in AUPR. The advantage is mainly due to our Mix-Decoder block that mitigates the above issue via its correction mechanism, introducing the binding site information and enhancing the interaction features. Moreover, our proposed feature fusion modules provide the model with more extensive and refined protein and drug features, effectively improving the performance of the model.

**Table 5.** A comparison with the state-of-the-art methods in terms of CI, MSE, $r_{m}^{2}$ index and AUPR, on the Davis and KIBA datasets, bold: best results

Dataset	Methods	CI↑	MSE↓	$r_{m}^{2}$ ↑	AUPR↑
Davis	DeepDTA (Öztürk *et al.*, 2018)	0.878 ± 0.004	0.261	0.630 ± 0.017	0.714 ± 0.010
	CPInformer (Hua *et al.*, 2022)	0.874 ± 0.002	0.277	0.618 ± 0.004	0.663 ± 0.003
	DeepCDA (Abbasi *et al.*, 2020)	0.891 ± 0.003	0.248	0.649 ± 0.009	0.739 ± 0.006
	MATT-DTI (Zeng *et al.*, 2021)	0.890 ± 0.003	0.229	0.682 ± 0.009	—
	GraphDTA (GIN) (Nguyen *et al.*, 2021)	0.890 ± 0.005	0.233 ± 0.004	0.663 ± 0.010	0.725 ± 0.005
	DeepGLSTM (Mukherjee *et al.*, 2022)	0.893 ± 0.003	0.236 ± 0.004	0.677 ± 0.006	0.719 ± 0.006
	MFR-DTA	**0.905 ± 0.001**	**0.221 ± 0.001**	**0.705 ± 0.003**	**0.751 ± 0.003**
KIBA	DeepDTA (Öztürk *et al.*, 2018)	0.863 ± 0.002	0.194	0.673 ± 0.009	0.788 ± 0.004
	CPInformer (Hua *et al.*, 2022)	0.867 ± 0.003	0.183	0.677 ± 0.002	0.773 ± 0.004
	DeepCDA (Abbasi *et al.*, 2020)	0.889 ± 0.002	0.176	0.682 ± 0.008	0.812 ± 0.005
	MATT-DTI (Zeng *et al.*, 2021)	0.889 ± 0.001	0.150	0.756 ± 0.011	—
	GraphDTA (GIN) (Nguyen *et al.*, 2021)	0.883 ± 0.004	0.151 ± 0.003	0.687 ± 0.010	0.775 ± 0.007
	DeepGLSTM (Mukherjee *et al.*, 2022)	0.890 ± 0.004	0.143 ± 0.005	0.780 ± 0.006	0.783 ± 0.006
	MFR-DTA	**0.898 ± 0.002**	**0.136 ± 0.001**	**0.789 ± 0.002**	**0.827 ± 0.003**

Then, we evaluate MFR-DTA on the KIBA dataset, and the label distribution of the dataset is relatively normal. But most sample labels in this dataset are incredibly concentrated. Hence, it is difficult to predict the affinity trend, hindering most models from performing well in terms of the CI metric. However, the proposed MFR-DTA method still achieves 0.8% and 0.7% performance gains in terms of CI and MSE, respectively, as compared with the second-best method, DeepGLSTM. The main advantage of our method is the capability of discriminative biological sequence feature extraction, which can better distinguish the similarities and differences among samples, alleviating the issue associated with over-centralized labels. In general, the experiments obtained on both datasets demonstrate that our method, inheriting the minor standard deviation of the baseline method (Hua *et al.*, 2022), almost outperforms all the other methods in terms of all the evaluation metrics.

### 3.4 Visualization of drug–target BR prediction

In this part, we compare MFR-DTA with the existing approaches in predicting drug–target BRs. We take the probability of the actual binding site falling into the prediction region as the metric to measure the accuracy of these approaches. The prediction region length is *S* amino acid elements, and the midpoint is where with the highest value in the drug–target response vector mentioned in Section 2.3. We measure prediction regions of different methods according to three scales (*S*=5, 10 and 15) and report the evaluation results in Table 6. Obviously, the prediction accuracy of all the methods increases as the scale, *S*, increases. On Davis, all the existing methods perform poorly when *S* is smaller than 10, but the prediction of MTR-DTA is relatively more accurate. When *S*=15, the accuracy of MTR-DTA is 0.968, outperforming the other methods. On KIBA, all the methods are easier to predict the BR because the dataset has fewer protein types but more samples. However, MTR-DTA still performs better than the others. Besides, we supply a new dataset, sc-PDB (Gaber *et al.*, 2019), that includes actual BR information, to evaluate these models in predicting unseen drug–target BRs with the scale *S*=15. The experimental results also confirm that the performance of a BR prediction model with supervised learning is more reliable. Note that the prediction performance of the model trained on Davis is better than that trained on KIBA. This is mainly due to the diversity of proteins and a wider spectrum of drugs in the Davis dataset, as shown in Figure 3. This also illustrates that the performance of a model in predicting unseen drug–targeted BRs could be further improved with the selection of a training set. In fact, it is unfair to compare the proposed model with these unsupervised approaches directly. However, this experiment reveals that training a model with the prior-binding-site knowledge improves the prediction accuracy significantly.

**Table 6.** A comparison of different methods in predicting the drug–target BRs, evaluated in terms of accuracy, bold: best results

Dataset	Methods	*S*=5	*S*=10	*S*=15	sc-PDB
Davis	CPI-GNN	0.073	0.126	0.201	0.081
	DeepCDA	0.166	0.257	0.339	0.191
	TransformerCPI	0.085	0.213	0.289	0.237
	CPInformer	0.101	0.158	0.318	0.213
	MTR-DTA	**0.174**	**0.459**	**0.968**	**0.621**
KIBA	CPI-GNN	0.191	0.313	0.391	0.093
	DeepCDA	0.325	0.472	0.501	0.151
	TransformerCPI	0.287	0.374	0.419	0.196
	CPInformer	0.301	0.488	0.529	0.218
	MTR-DTA	**0.651**	**0.902**	**0.942**	**0.513**

**Fig. 6.**
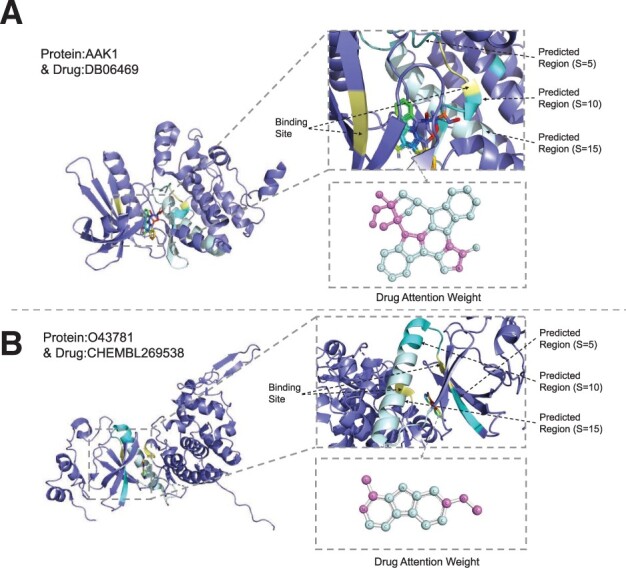
Visualization of the predicted BRs at three scales and binding sites in two sample pairs: (**A**) protein ‘AAK1’ and drug ‘DB06469’ and (**B**) protein ‘O43781’ and drug ‘CHEMBL269538’

We also visualize the test samples proposed in Section 1 to further demonstrate the effectiveness of the proposed method. As shown in Figure 6, we magnify the interaction part to the right side of the figure. In this part, the blue-purple region is the non-interaction region. The light blue, blue and dark blue regions are the prediction regions with *S*=15, 10 and 5, respectively. The yellow position is the actual binding site. It is clear that the binding sites of the protein ‘O43781’ accurately fall into the predicted region with the scale, S≥ 5. While the prediction of the protein ‘AAK1’ is relatively biased, but the binding site can also fall in the region with the scale, S≥ 10. It is intuitive to show the excellent performance of our method for predicting BRs. Meanwhile, we visualize the drug molecules according to the feature S-E [Equation (9)] parameter $f_{a}(X)$, in which the pink region represents a higher attention weight. Unfortunately, similar to other approaches, this visualization only reflects where the computer pays attention without any biomedical meaning. To migrate this deficiency, in our future work, we will further explore the functional regions of the drug molecule that act on the protein to enhance the biomedical interpretability of a model.

## 4 Conclusion

We presented a novel MFR-DTA method to predict DTA and region simultaneously. We first extracted biological sequence features via the BioMLP/CNN block, integrating individual element features and global position features. Then, we fused and refined the extracted features by the Elem-feature fusion block. Afterwards, we developed Mix-Decoder to extract the DTI features for BR prediction. Last, we predicted DTA by applying fully connected layers to the interaction features. The experimental results obtained on three datasets verified the superiority of our method over the state-of-the-art approaches. Besides, we visualized some samples to present the positional relationships between binding sites and predicted multi-scale interaction regions. However, our visualization of drug molecules is still based on attention weights. Therefore, we will further explore the structural factors of drug molecules acting on proteins in our future studies on DTA prediction. Meanwhile, we aim to collect a new dataset with a wider spectrum of proteins and chemical drugs to improve the robustness of a deep learning model. Generally, we will continue to enhance the biological interpretability of the DTA model while improving its accuracy to assist biomedical researchers in discovering new drugs.
